# Impact of obstructive sleep apnea and comorbid insomnia on all-cause mortality: a prospective cohort study of 2401 patients with 6-year follow-up

**DOI:** 10.1007/s44470-026-00068-w

**Published:** 2026-04-08

**Authors:** Sahrai Saeed, Ragnhild Stokke Lundetræ, Andrea Romarheim, Ingvild West Saxvig, Sverre Lehmann, Bjørn Bjorvatn

**Affiliations:** 1 Department of Heart Disease, Haukeland University Hospital, Jonas Lies Vei 65, 5021 Bergen, Norway; 2 Department of Cardiology, Ullevaal & Faculty of Medicine, Oslo University Hospital, University of Oslo, Oslo, Norway; 3 Department of Global Public Health and Primary Care, University of Bergen, Bergen, Norway; 4 Department of Internal Medicine, Voss Hospital, Voss, Norway; 5 Norwegian Competence Center for Sleep Disorders, Haukeland University Hospital, Bergen, Norway; 6 Department of Thoracic Medicine, Haukeland University Hospital, Bergen, Norway; 7 Department of Clinical Science, University of Bergen, Bergen, Norway

**Keywords:** Respiratory event index, Hypertension, Comorbid insomnia and sleep apnea, Obstructive sleep apnea

## Abstract

**Study Objectives:**

The impact of obstructive sleep apnea (OSA) on all-cause mortality needs further research as earlier data are inconclusive. Between 30 and 50% of patients with OSA have comorbid insomnia (COMISA), which may increase the risk of morbidity and mortality. The present study was aimed at investigating the association between OSA and COMISA and the subsequent risk of all-cause mortality in a cohort of patients recruited from a hospital sleep clinic setting.

**Methods:**

Between January 2016 and December 2018, 2401 patients with suspected OSA underwent standard respiratory polygraphy and were recruited in the present analysis. OSA was categorized according to the respiratory event index (REI). Insomnia diagnosis was assessed with the Bergen Insomnia Scale. All-cause mortality was the primary endpoint.

**Results:**

Mean age was 49.6 ± 14.0 years, 68.8% were males, and 36.2% had OSA with REI ≥ 15, 49.5% insomnia, and 16.9% COMISA (comorbid insomnia and OSA with REI ≥ 15). A progressive increase in the risk of all-cause mortality was observed with increasing OSA severity (REI < 5; 5–14.9; 15–29.9; ≥ 30). In a multivariable Cox regression analysis, OSA (REI ≥ 15) was independently associated with all-cause mortality (HR 2.65; 95% CI 1.12–6.30, *p* = 0.027). This relative risk was further increased to threefold (HR 3.02; 95% CI 1.30–7.04, *p* = 0.010) when OSA was replaced by COMISA in the same model.

**Conclusion:**

This study demonstrates that OSA severity is an important determinant of long-term survival. Patients with moderate-to-severe OSA are at particularly increased risk, and the coexistence of insomnia appears to substantially amplify this risk. These findings underscore the prognostic significance of considering both sleep-disordered breathing and comorbid insomnia when assessing mortality risk in patients evaluated for OSA.

**Brief summary:**

Obstructive sleep apnea (OSA) is linked to cardiovascular disease, but its impact on mortality, especially when combined with insomnia (COMISA), remains underexplored. This study investigated the prognostic OSA severity and COMISA in a large cohort of 2401 patients with suspected OSA. Our findings showed that moderate to severe OSA significantly predicted all-cause mortality, independent of traditional risk factors. The presence of COMISA tripled this risk, highlighting a synergistic negative effect. These results emphasize the importance of recognizing COMISA as a distinct, high-risk phenotype. Future clinical practice should include systematic screening and treatment of both OSA and insomnia to improve survival outcomes in affected patients.

## Introduction

Obstructive sleep apnea (OSA), caused by recurrent collapse of upper airways during sleep with subsequent episodes of apnea/hypopnea, is a common sleep-related breathing disorder and a major public health concern [1]. OSA is associated with an increased risk of cardiovascular events through several possible mechanisms such as coronary artery disease, atrial fibrillation, intermittent hypoxia causing elevated blood pressure and functional and structural remodeling of the heart and arterial tree, sleep fragmentation, and intra-thoracic pressure fluctuations [1, 2]. In one of our previous studies of 6500 participants with suspected OSA, we confirmed the diagnosis of OSA in 35% of the total sample [2]. Patients with OSA were more likely to be male and had higher rates of modifiable cardiovascular risk factors including hypertension and obesity compared to no OSA controls (REI < 15/h) [2]. However, the prognostic significance of OSA is less explored in such large-scale studies. Overall, there have been few studies in the literature investigating the impact of OSA on mortality, and data on the association between OSA and the risk of cardiovascular events and mortality are inconsistent [3–7]. Meta-analyses assessing the association of OSA and cardiovascular outcomes and mortality have also some limitations although their sensitivity analyses indicated robust results [4, 6, 7]. Furthermore, OSA commonly co-occur with insomnia, a sleep disorder characterized by sleep initiation or maintenance problems and subsequent daytime impairments [8]. OSA and insomnia appear to share a bidirectional association and to even exacerbate each other [9, 10]. Compared to lone insomnia or OSA, comorbid insomnia and sleep apnea (COMISA) is associated with a greater risk of morbidity and mortality [11]. However, there have been few research studies to investigate the prevalence, severity, natural history, and prognostic impact of COMISA across sex, race, and age. Hence, to further expand the abovementioned findings, in the present paper, we aimed to investigate the association between OSA and COMISA and the subsequent risk of all-cause mortality in our large database of patients with sleep-related breathing disorders recruited from a hospital sleep clinic setting.

## Methods

### Study design and participants

The study population comprised 2401 consecutive patients with suspected OSA who were referred to the Center for Sleep Medicine at Haukeland University Hospital, Bergen between January 2016 and December 2018. Data collection and methodology have previously been described in detail [2]. OSA was assessed using standard respiratory polygraphy, and scored using the 4% oxygen desaturation criteria for hypopneas [12]. Classification of obstructive sleep apnea (OSA) can be based on either the apnea–hypopnea index (AHI) or the respiratory event index (REI). In this study, the REI was used to determine the presence and severity of OSA, defined as the number of apneas and hypopneas per hour of monitoring. OSA was categorized as follows: no OSA (REI < 5), mild OSA (REI 5–14.9), moderate OSA (REI 15–29.9), and severe OSA (REI ≥ 30) [13]. The self-reported questionnaire included information on comorbid medical conditions such as previous myocardial infarction, angina pectoris, stroke, hypertension, diabetes, chronic obstructive pulmonary disease (COPD), and asthma. Symptoms of insomnia were assessed by the validated Bergen Insomnia Scale (BIS) [13]. The BIS consists of six items scored along an eight-point scale indicating the number of days per week (0–7 days) for which a specific insomnia symptom is experienced. Chronic insomnia was defined according to the updated ICSD-3 and DSM-5 as scoring ≥ 3 days per week on at least one of the first three items of the scale (sleep latency exceeding 30 min, wake after sleep onset exceeding 30 min, and early morning awakening exceeding 30 min), in addition to ≥ 3 days per week on at least one of the two latter items (daytime impairment and dissatisfaction with sleep) [14, 15]. Of note, until 2018, BIS addressed symptoms during the past month, while from 2018 and onwards, the scale addressed symptoms during the past 3 months.

All patients completed a comprehensive questionnaire that included information on medical comorbidities, the Epworth Sleepiness Scale, current smoking habits (number of cigarettes per day), and alcohol consumption (number of units and frequency). The smoking variable (number of cigarettes per day) was dichotomized into no smoking and current smoking (if one or more cigarettes per day). Excessive alcohol consumption was defined as alcohol intake on ≥ 3 days per week. During the consultation, the patients’ weight and height were measured and body mass index (BMI) was calculated by weight in kg divided by squared height in meters. Obesity was defined as BMI ≥ 30 kg/m^2^. Blood pressure was measured by a nurse or a doctor after a 10-min rest in the sitting position by an automatic device (OMRON or Scan-Med). In the case of strongly deviating values, blood pressure was remeasured manually. Hypertension was defined as previous history of hypertension, use of antihypertension medications, or elevated clinic systolic BP (≥ 140 mmHg) and/or diastolic BP (≥ 90 mmHg) [16]. Cardiovascular disease was defined as stroke and/or myocardial infarction.

### Endpoints

All-cause deaths were the endpoints of interest and were recorded during follow-up by reviewing the electronic patient record or death certificates with censoring date 15.04.2023. The information about insomnia was missing in 293 patients, resulting in 2108 patients for inclusion in the survival analyses of COMISA versus No-COMISA groups.

### Data analysis

SPSS (IBM Corporation, Armonk, New York, USA) version 28 was used for the data analyses. Continuous variables are presented as mean ± standard deviation and categorical variables in percentage. Survival estimates were examined by Kaplan–Meier analyses, and the difference between groups was tested using a log-rank test. Cox proportional hazard models were used to assess the impact of OSA and COMISA on all-cause mortality. All predictors of mortality with statistically significant association in univariate Cox regression analyses or clinically relevant (male sex and BMI) were included in the multivariate Cox regression models. Three different multivariate Cox models were constructed for each OSA, COMISA by REI ≥ 5, and COMISA by REI ≥ 15. Smoking and chronic obstructive pulmonary disease were not included in the same model because of collinearity. A *p*-value < 0.05 was considered statistically significant.

## Results

**Fig. 1:**
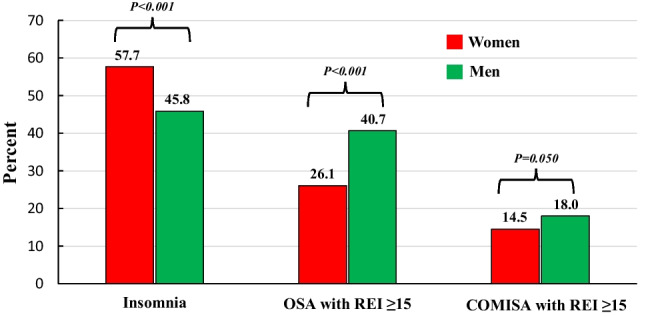
The prevalence of insomnia, obstructive sleep apnea (OSA), and comorbid insomnia and sleep apnea (COMISA) in women and men

Baseline demographics and clinical characteristics are presented in Table 1. Mean age was 49.6 ± 14.0 years, 68.8% were males, and 36.2% had OSA with REI ≥ 15, 49.5% insomnia, and 16.9% COMISA (comorbid insomnia and OSA with REI ≥ 15/h). The prevalence of hypertension was 55.6% and obesity 44.0%. Among patients with moderate-to-severe OSA (REI ≥ 15), 46.7% had insomnia, while the prevalence of OSA in patients with insomnia was 34.2%. The prevalence of insomnia was 57.7% in women and 45.8% in men (*p* < 0.001), while the prevalence of OSA with REI ≥ 15 was 26.1% in women and 40.7% in men (*p* < 0.001) (Fig. 1). There was no difference in the prevalence of COMISA with REI ≥ 5 within the sexes (women 33.8% and men 33.5%, *p* = 0.867), while a trend towards a higher proportion of patients with COMISA by REI ≥ 15 in men compared with women (18.0% vs 14.5%, *p* = 0.050) was observed. The prevalence of excessive sleepiness was 33.5% in patients with no OSA and no insomnia, 36.3% in OSA alone, 38.4% in insomnia alone, and 49.0% in the COMISA group (*p* < 0.001). Demographics, and the frequencies of cardiovascular morbidities and all-cause mortality across OSA–Insomnia subtypes are presented in Table 2. An increase was observed in age and the prevalence of obesity, CVD, and all-cause mortality across groups.

**Table 1:** Baseline characteristics of the total study population

	Total population (*n* = 2401) Mean ± SD or *n* (%)
Age (years)	49.6 ± 14.0
Male sex, *n* (%)	1651 (68.8)
Body mass index (kg/m^2^)	30.1 ± 6.9
Obesity (body mass index ≥ 30 kg/m^2^), *n* (%)	1056 (44.0)
Current smoking, *n* (%)	463 (19.3)
Alcohol consumption ≥ 3 d/week, *n* (%)	166 (6.9)
Serum creatinine (μmol/L)	79 ± 22
Systolic blood pressure (mmHg)	133 ± 15
Diastolic blood pressure (mmHg)	81 ± 10
Hypertension, *n* (%)	1343 (55.6)
Cardiovascular disease, *n* (%)	178 (7.4)
Diabetes, *n* (%)	201 (8.4)
Chronic lung disease^*^, *n* (%)	456 (19.0)
REI per hour sleep	16.0 ± 17.7
OSA according to REI categories, *n* (%)	
REI < 5 REI 5–14.9 REI 15–29.9 REI ≥ 30	731 (30.4) 802 (33.4) 482 (20.2) 384 (16.0)
Excessive sleepiness (Epworth Sleepiness Scale = 11 or higher), *n* (%)	888 (37.0)
Insomnia, *n* (%)	1043 (49.5)
Comorbid insomnia and OSA with REI ≥ 5 (%)	708 (33.6)
Comorbid insomnia and OSA with REI ≥ 15 (%)	357 (16.9)
Follow-up time (months)	69.1 ± 11.6

*REI* respiratory event index

^*^Chronic obstructive pulmonary disease and/or asthma

**Table 2:** Demographics and the frequencies of cardiovascular morbidities and all-cause mortality across OSA–Insomnia subtypes

	No OSA, no insomnia (*n* = 657)	Insomnia alone (*n* = 686)	OSA alone (*n* = 408)	COMISA (*n* = 357)	*p* value
Age (year ± SD)	46.4 ± 14.0	47.3 ± 13.9	54.8 ± 12.5	55.0 ± 11.8	< 0.001
Male sex (%)	68.5	58.9	83.1	73.4	< 0.001
Obesity (%)	34	38	57	59	< 0.001
Established CVD (%)	5.2	6.3	10.0	11.2	< 0.001
All-cause mortality, *n* (%)	14 (2.1)	14 (2.0)	13 (3.2)	22 (6.2)	< 0.001

*CVD* cardiovascular disease, *COMISA* comorbid insomnia and sleep apnea, *OSA* obstructive sleep apnea, *OSA* REI ≥ 30

**Fig. 2:**
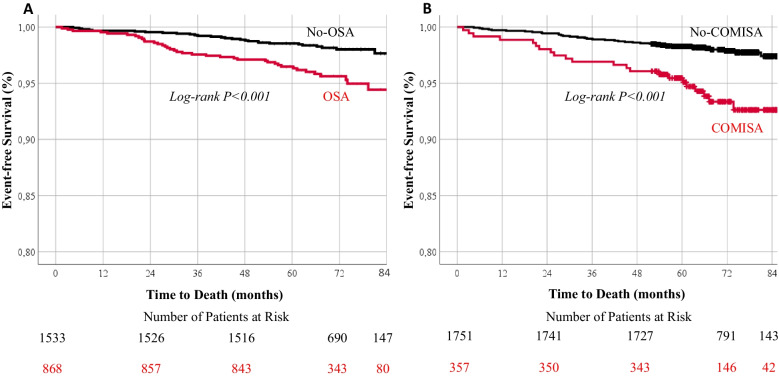
Kaplan–Meier plots showing the cumulative probability of all-cause mortality according to OSA (REI < 15 versus REI ≥ 15) (**A**) and COMISA (**B**). *OSA*, obstructive sleep apnea; *COMISA*, comorbid insomnia and sleep apnea; *REI*, respiratory event index

**Fig. 3:**
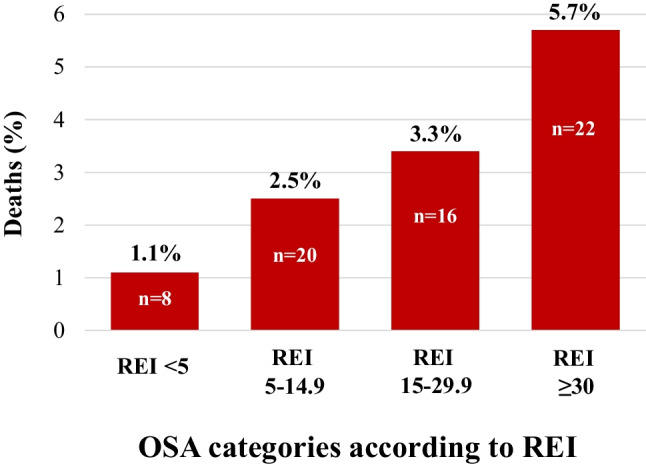
Rates of all-cause mortality across the OSA severity by REI. *REI*, respiratory event index; *OSA*, obstructive sleep apnea

During a mean follow-up of 5.8 ± 1.0 years, 66 deaths occurred. Patients with moderate to severe OSA (REI ≥ 15) and COMISA had significantly reduced survival compared with No-OSA (REI < 15) and No-COMISA patients (Fig. 2). Mean REI was 16 ± 17 in patients who were alive (*n* = 2335) compared to 24 ± 18 in those who deceased (*n* = 66) (*p* < 0.001). There was a progressive increase in the risk of all-cause mortality with increasing OSA severity (Fig. 3, Table 3). In a multivariable Cox regression analysis adjusting for age, sex, BMI, serum creatinine, hypertension, cardiovascular disease, diabetes and chronic lung disease, moderate to severe OSA was independently associated with all-cause mortality (hazard ratio [HR] 2.65; 95% CI 1.12–6.30, *p* = 0.027) (Table 3). COMISA with REI ≥ 5 was a strong predictor of all-cause mortality in a univariate Cox regression analysis (Table 3). In a secondary multivariable model adjusting for the same covariates as shown in Table 3, but replacing OSA by COMISA (comorbid insomnia and OSA with REI ≥ 5), COMISA was still associated with all-cause mortality (HR 2.41; 95% CI 1.04–5.57, *p* = 0.040), independent of significant association with age (HR 1.07; 95% CI 1.03–1.11, *p* = 0.001), creatinine (HR 1.01; 95% CI 1.00–1.01, *p* = 0.025), and chronic lung disease (HR 2.34; 95% CI 1.04–5.30), *p* = 0.041) and non-significant associations with sex, BMI, hypertension, cardiovascular disease, and diabetes (all *p* > 0.05). Furthermore, when COMISA was defined by REI ≥ 15, it was COMISA (HR 3.02; 95% CI 1.30–7.04, *p* = 0.010), age (HR 1.07; 95% CI 1.03–1.11, *p* = 0.001) and creatinine (HR 1.01; 95% CI 1.00–1.01, *p* = 0.035), and not chronic lung disease (HR 2.23; 95% CI 0.98–5.01, *p* = 0.057), which were associated with all-cause mortality, independent of no-significant associations with sex, BMI, hypertension, cardiovascular disease, and diabetes (all *p* > 0.05).

**Table 3:** Predictors of all-cause mortality in univariate and multivariate Cox regression analyses in the entire study population (*n* = 2401)

	Univariate		Multivariate	
	HR (95% CI)	*p*	HR (95% CI)	*p*
Age (year)	1.10 (1.08–1.12)	< 0.001	1.07 (1.03–1.11)	0.001
Male sex	1.32 (0.76–2.29)	0.328	0.97 (0.41–2.27)	0.936
BMI (kg/m^2^)	0.99 (0.96–1.03)	0.803	0.99 (0.92–1.07)	0.789
Obesity (BMI ≥ 30 kg/m^2^)	1.14 (0.71–1.85)	0.589		
Smoking	2.35 (1.39–3.96)	0.001		
Serum creatinine (μmol/L)	1.01 (1.00–1.01	< 0.001	1.00 (0.99–1.01)	0.150
Systolic BP (mmHg)	1.00 (0.98–1.02)	0.925		
Diastolic BP (mmHg)	0.98 (0.96–1.00)	0.081		
Pulse pressure (mmHg)	1.01 (0.99–1.03)	0.176		
Pulse pressure ≥ 60 mmHg	1.56 (0.92–2.64)	0.098		
Hypertension	2.72 (1.53–4.84)	0.010	0.67 (0.28–1.61)	0.373
Cardiovascular disease	6.17 (3.68–10.36)	< 0.001	1.95 (0.80–4.74)	0.143
Diabetes	4.22 (2.38–7.48)	< 0.001	1.36 (0.60–3.07)	0.464
Chronic lung disease^*^	2.92 (1.70–5.01)	< 0.001	2.27 (1.03–4.97)	0.041
OSA with REI ≥ 15 vs REI < 15	2.46 (1.51–4.00)	< 0.001	2.65 (1.12–6.30)	0.027
OSA with REI ≥ 5 vs no OSA (REI < 5)	3.36 (1.60–7.03)	0.001		
Mild OSA (REI 5–14.9) vs no OSA (REI < 5)	2.29 (1.01–5.19)	0.048		
Moderate OSA (REI 15–29.9) vs no OSA (REI < 5)	3.05 (1.30–7.11)	0.010		
Severe OSA (REI ≥ 30) vs no OSA (REI < 5)	5.51 (2.45–12.38)	< 0.001		
Insomnia	1.68 (0.99–2.85)	0.056		
Comorbid insomnia and OSA with REI ≥ 5	3.06 (1.81–5.18)	0.015	2.41 (1.04–5.57)	0.040
Comorbid insomnia and OSA with REI ≥ 15	3.09 (1.82–5.26)	< 0.001	3.02 (1.30–7.04)	0.007

*BMI* body mass index, *BP* blood pressure, *COPD* chronic obstructive pulmonary disease, *OSA* obstructive sleep apnea, *REI* respiratory event index

^*^COPD and/or asthma

## Discussion

In this large-scale prospective cohort study of 2401 patients with suspected OSA who were referred for diagnostic assessment at a tertiary sleep center, the prevalence of OSA was 36.2%, insomnia 49.5%, and COMISA (comorbid insomnia and OSA with REI ≥ 15) 16.9%.

We found an incremental increase in the risk of all-cause mortality with increasing OSA severity. Moderate or severe OSA (REI ≥ 15) had a strong and independent association with all-cause mortality, and this effect was further increased to threefold if OSA patients also had comorbid insomnia (COMISA) (see later). Other predictors of mortality were age, creatinine, and chronic lung disease.

The relationship between OSA and cardiovascular mortality is dependent on the severity of OSA. Severe OSA (REI ≥ 30) was associated with reduced survival whereas milder form of OSA was not [4]. Other studies have also indicated that severe OSA significantly increases the risk of cardiovascular and all-cause mortality [3–5]. A meta-analysis by Xie et al., based on 16 prospective cohort studies and 24,308 individuals, concluded that moderate and severe OSA, but not mild OSA, was associated with an increased risk of vascular outcomes and all-cause mortality [7]. However, the authors speculated that this relationship might differ between sexes. In the present study, we show that in univariate Cox regression analysis, sex had no association with all-cause mortality, and when sex, due to its clinical relevance, was introduced into the multivariable model, OSA and COMISA were still strongly and independently associated with all-cause mortality. In another meta-analysis of 13 hospital-based cohort studies, the authors investigated the relationship between OSA and recurrent vascular events and all-cause mortality. However, the investigators compared the highest REI with the lowest REI, and the grade of OSA severity and subsequent adverse outcomes were not assessed [7]. Furthermore, most of the relative risks were calculated by the authors and not adjusted for traditional cardiovascular risk factors, as these estimates were not reported in the original studies.

### Comorbid insomnia and sleep apnea (COMISA)

Insomnia, similar to OSA, is a prevalent debilitating sleep disorder that is associated with reduced quality of mental and physical health and higher use of health resources [17]. COMISA has a dynamic interplay with bidirectional associations. Insomnia may enhance ventilatory instability, increase loop gain, or interfere with sleep-dependent compensatory mechanisms in OSA. Conversely, untreated OSA may perpetuate chronic hyperarousal and cortical activation, making insomnia more resistant to therapy. In our study, the prevalence of insomnia was 46.7% in OSA with REI ≥ 15, while the prevalence of OSA in patients with insomnia was 34.2%. These estimates are perfectly in line with those presented in other studies showing that overall, approximately 30–40% of people with insomnia have co-morbid sleep apnea, and 30–50% of people with sleep apnea have co-morbid insomnia [17].

Previous studies have demonstrated that insomnia is related to the higher mortality risk in the general population or patients [18–20]. Most recently, data from UK biobank including 35,757 individuals (mean age 61.2 ± 6.3 years) with cardiometabolic multimorbidity, not having insomnia was independently associated with an 8% lower risk of all-cause mortality [21]. On the other hand, there are also meta-analyses of prospective cohort studies that have reported no associations between insomnia-alone and mortality [22, 23].

However, the incremental increase in the risk of all-cause mortality in COMISA compared to OSA alone is not fully understood.

COMISA was initially described by Guilleminault et al. a half-century ago [24], but the term was coined only in 2017 in the medical literature, which since then received growing attention. Three recent population-based cohort studies have shown an association between COMISA and all-cause mortality over a 10–20 year follow-up [11, 25, 26]. In our study, we confirmed these findings. However, compared to the adjusted hazard ratio reported by the previous studies (HR 1.47, 1.57, and 1.71) [11, 25, 26], the adjusted hazard ratio for all-cause mortality in our COMISA patients was nearly twice as high (HR 3.02) as the ratio among patients with no-COMISA. This may be explained by the fact that despite a comparable age, the prevalence of cardiometabolic risk factors such as hypertension, obesity, and diabetes was lower in the above-mentioned studies [11, 25, 26]. Additionally, differences in the methodology for defining insomnia and OSA categories between studies might be other contributing factors. It is also likely that there are differences in the risk of mortality in people referred to a hospital sleep clinic, compared to population-based cohorts. This could explain the increased risk of mortality vs controls in the present study compared with the previous population-based cohorts. Finally, subgroup analyses, particularly for COMISA-INSD (Co-Morbid Insomnia and Sleep Apnea—Insomnia Disorder with Normal Sleep Duration), were based on relatively small numbers, resulting in wide confidence intervals and limited precision. Consequently, estimates may be vulnerable to sparse-data bias and reduced statistical power. The lack of statistically significant associations in COMISA-INSD may therefore reflect type II error rather than true absence of effect. These findings should be interpreted cautiously and require replication in larger samples with greater representation of COMISA subtypes.

### Strengths and limitations

The relatively large sample size of patients recruited from a hospital sleep clinic setting provided sufficient statistical power to the analyses. Furthermore, the sample comprised patients referred to a university hospital for suspicion of OSA from a wider geographic area in Western Norway, which is representative for the overall OSA population and their comorbid conditions, increasing the chance of generalizability of our findings. All patients who were diagnosed with OSA were assigned for a continuous positive airway pressure (CPAP) treatment. However, information about the use of CPAP or adherence to CPAP was not available, and our multivariate models could therefore not be adjusted for this unmeasurable factor, which is a limitation of our study. Another possible limitation is that insomnia was assessed by questionnaire and not a clinical interview. Hypertension was defined as a history of hypertension, use of antihypertension medications, or elevated clinic BP. Antihypertensive medication use captures only those who are diagnosed and treated, missing undiagnosed, untreated, or inconsistently treated individuals. Therefore, using medication use alone may underestimate the true burden of hypertension, which is better assessed by combining measured blood pressure (clinic and 24-h ambulatory), self-reported diagnosis, and treatment status. Furthermore, information about the duration of antihypertensive treatment was not known. Although the BIS is a validated instrument and very sensitive to insomnia, it is not highly specific in that it does not rule out the possibility that symptoms may be better explained by other sleep disorders. Moreover, the time frame in BIS was changed from complaints during the past month (applied in the current study) to the past 3 months in 2018, according to the new diagnostic criteria in DSM-5.

The clinical significance and prognostic implications of insomnia phenotypes by objective sleep duration are currently the focus of sleep research. Recent studies have shown that insomnia with objectively short sleep duration (polysomnography-measured total sleep time < 6 h) is more strongly related to incident hypertension in adults [27] and cardiovascular and/or cerebrovascular disease [28], and mortality [29] than insomnia with normal sleep duration. Indeed, insomnia with short sleep duration may act synergistically with OSA via shared mechanisms such as hypothalamic–pituitary–adrenal (HPA) axis overactivity, inflammation, and sympathetic overdrive, thereby amplifying mortality risk. Although the current study uses the BIS, it lacks data to discuss insomnia phenotypes based on objective or estimated sleep duration, as well as their prognostic impact. Hence, future studies should stratify COMISA not only by REI thresholds but also by sleep duration-based insomnia phenotypes to enhance risk stratification. When objective sleep duration is derived from a single-night PSG, estimates may be influenced by first-night effects (systematic alterations in sleep pattern and duration that occur when individuals sleep in a novel environment for the first time), mismatches between fixed laboratory sleep opportunities, and habitual sleep schedules. Individuals whose habitual sleep window is shorter or later may appear to have reduced objective sleep duration simply because the recording window does not match their biological or behavioral sleep timing. In this regard, sleep latency may be artificially prolonged, and total sleep time may be underestimated relative to habitual sleep. The important implications for these observations are that objective sleep duration becomes a function—not only of sleep ability but also of schedule misalignment. Taken together, these factors may lead to systematic underestimation of objective sleep duration, particularly those characterized by heightened arousal or irregular sleep timing. Furthermore, the current analytical approach treats COMISA as a single, binary entity. However, evidence supports that there is phenotypic heterogeneity of COMISA, reflecting different etiologies and risk trajectories. For instance, the association between OSA and insomnia may be bidirectional, i.e., some patients experience insomnia as a secondary consequence of OSA, mediated by arousals and sleep fragmentation, while others may develop OSA on a background of chronic insomnia and hyperarousal, with instability in ventilatory control possibly emerging downstream of insomnia. In contrast, a third group may represent a distinct phenotype, where insomnia and OSA co-occur due to shared predispositions (e.g., autonomic dysregulation, circadian misalignment, and comorbid psychiatric vulnerability). Addressing the phenotypic heterogeneity of COMISA and direct subgroup analyses could potentially strengthen the conceptual and clinical relevance of the current work. Clinically, the different OSA phenotypes, such as REM-related, positional, or non-sleep-stage-specific OSA, may interact variably with insomnia features. REM-related OSA, for instance, may lead to morning awakenings, while supine-predominant OSA may present with more fragmented sleep, enhancing insomnia symptoms. Although our study did not directly assess OSA phenotypes, it is plausible that the type and timing of apneic events modulate how insomnia contributes to physiological stress and, ultimately, mortality. In other words, the COMISA–mortality link may not be uniform but vary by OSA phenotype through differential effects on autonomic activation, sleep architecture, and treatment responsiveness. In our study, the no OSA group is composed of patients who were referred for suspected OSA and reported multiple comorbidities and sleep complaints; therefore, this group does not represent a healthy general population. Finally, compared to polysomnography, polygraphy monitors the number of apneas/hypopneas per monitoring hour and commonly underestimates the number of obstructive respiratory events per hour. Furthermore, the possibility of false negative polygraphy results may underestimate the true prevalence of COMISA. This may explain the sex-disparity regarding men having a higher frequency of COMISA when OSA was defined as REI ≥ 15 and no differences between the sexes using REI ≥ 5.

### Future directions

Patients with sleep-related breathing disorders referred for OSA assessment should be systematically screened for comorbid insomnia, and if confirmed, both conditions should be treated optimally to reduce the risk of future vascular events and mortality. Although our findings strongly support screening for COMISA in clinical settings, it is unlikely that a single treatment strategy will be effective for all cases. For instance, patients with insomnia-driven COMISA may benefit most from Cognitive Behavioral Therapy for Insomnia (CBT-I) first, followed by PAP (positive airway pressure) therapy to improve sleep quality, adherence, and overall treatment outcomes. Conversely, OSA-predominant COMISA may respond better to early PAP therapy, with subsequent insomnia re-evaluation. A personalized phenotyping and risk stratification phenotype-based therapeutic strategies may improve both symptom resolution and survival. Larger, well-designed, prospective research studies in the future are warranted to elucidate the specific mechanisms and risk factors linking COMISA to mortality risk, as well as the effect of different therapeutic interventions to mitigate this harmful association.

## Conclusions

In our cohort of 2401 patients with suspected sleep-related breathing disorders who were referred for diagnostic assessment, 36.2% had OSA, 49.5% insomnia, and 16.9% COMISA (comorbid insomnia and OSA with REI ≥ 15/h). An incremental increase in the risk of all-cause mortality was found with increasing OSA severity. Moderate or severe OSA (REI ≥ 15) had a strong and independent association with all-cause mortality, and this effect was further increased to threefold if these patients also were suffering from comorbid insomnia (COMISA).

## Data Availability

Data is not openly accessed due to regulatory restrictions, but will be provided upon reasonable request from the corresponding author.

## Abbreviations

AHI: Apnea-hypopnea index
BIS: Bergen Insomnia Scale
BMI: Body mass index
BP: Blood pressure
COMISA: Comorbid insomnia and sleep apnea
COPD: Chronic obstructive pulmonary disease
CPAP: Continuous positive airway pressure
CVD: Cardiovascular disease
OSA: Obstructive sleep apnea
REI: Respiratory event index

## Declarations

### Clinical trial

The manuscript does not report on a clinical trial.
